# Employees and the National Insurance Act

**Published:** 1912-10-12

**Authors:** 


### NATIONAL INSURANCE ACT DEPARTMENT.

### EMPLOYEES AND THE NATIONAL INSURANCE ACT.

### XXX.—A Suggested Scheme for Domestic Servants.

In our issue of August 3 last we dealt at some length with the question of "Substituted Benefits." We pointed out that one of the principal failings of the State Scheme of Insurance was its too rigid adherence to a standard type, both for contributions and benefits, and that too little consideration was allowed for the varying needs of different classes of insured persons. We were able to show that some provision had been made in the Act with a view to finding a solution to the difficulty by allowing the substitution of benefits of the same actuarial value in place of the standard sickness and disablement allowances, or any part of either of them. The form to be taken by the substituted benefit was to be one or other of those set out in the schedule of additional benefits appended to the Act. This schedule of additional benefits was contained in the Bill as originally introduced into Parliament to provide the means of distributing surplus when this is disclosed by a valuation of a society.

The idea of substituting benefits to meet the special requirements of certain classes of insured persons was suggested by an amendment during the passage of the Bill through Committee, and was largely due to the agitation for the exclusion of domestic servants from the scope of the measure. Owing to the very small time allotted to the effective discussion of amendments, it was not realised that the additional benefits schedule, admirable though it might be for the purpose of distribution of surplus, might not exactly fulfil the requirements of substituted benefits. In point of fact the schedule is defective in several important particulars, and, although a special committee was appointed by the Insurance Commissioners to consider the whole question of substituted benefits, the sole recommendation of that committee was a scheme for deferred pensions, commencing at the age of sixty-five, in place of the sickness and disablement allowances.

### Ineffectiveness of the Official Scheme.

So little faith had the committee in their own suggestion that the chairman, Mr. C. F. G. Masterman, in a special introduction to their report, thought it necessary to emphasise in the strongest possible terms that the substitution could only be allowed in the case of certain special classes of employed persons, and that even amongst such classes it would be a matter for serious consideration by each individual whether the benefits of the Act were not more advantageous.

We showed in our issue of August 3 that in the most favourable circumstances the amounts of deferred pension obtainable were quite small and hardly worth taking, especially in the case of women. Furthermore, the substitution of a deferred pension for a sickness benefit would entirely alter the character of the insurance, and thus to a large extent defeat the object of the scheme of National Insurance.

The official scheme, therefore, stood condemned as it substituted a benefit, which, both in form and substance, was no better than—if it was as good as—the benefit it was designed to replace. So far as we have been able to gather, the Commissioners have not yet approved the adoption of their official scheme by any approved society, and the matter of meeting the special requirements of insured persons is more or less where it stood upon the passing of the Act.

### The Special Needs of Domestic Servants.

The principal class of insured persons to whom the sickness benefit, as provided under the Act, is not suitable is that of domestic servants, and, as we have already indicated, it was chiefly on their behalf that the amendment was introduced and finally incorporated in the Act.

It is a matter of common knowledge that domestic servants, as a class, are treated with care and attention by their mistresses during short periods of illness, and it is somewhat of an anomaly that by the operation of the Act a domestic servant will be in a better financial position when she is ill than when she is well, at least so far as short illnesses are concerned. On the one hand, the Act prescribes that the receipt of sickness benefit shall not be a ground for dismissing a servant otherwise than with the usual notice or wages in substitution thereof; and, on the other hand, it is laid down that the benefits are inalienable. Thus the mistress is not relieved of her obligation to pay wages while the servant is ill, and the servant cannot enter into an agreement whereby the sick pay could be claimed by the mistress as compensation for the loss and trouble she may be occasioned through the illness.

It is easy to see that the position is one in which abuse could readily be made of the insurance, and it is strange that the matter was not realised in time for due safeguards to be inserted in the Act. These safeguards are as necessary from the point of view of the mistress as of the approved societies, for the extra trouble of nursing a sick servant and the expense that may be incurred in the employment of other domestic assistance is not a prospect that can be viewed with unconcern if it can be prevented. Malingering on any large scale would immediately have a serious prejudicial effect on the finances of an approved society undertaking this class of risk, and it would be very hard to make the inspection of claims so rigid as entirely to exclude all but *bona-fide* cases.

Here then is the position. The sickness benefit as provided by the Act is shown to be unnecessary to a large body of domestic servants during short periods of illness, whilst from the points of view of the mistresses and societies there are valid reasons for desiring its alteration. The only official alteration that has been suggested is lacking in essential qualities, and no amendment of the Act is possible except by means of an amending Act, and it is unthinkable that such an Act could be passed through Parliament in the present over-burdened session to meet the particular difficulty, without raising other matters which also claim amendment, and thus risking the failure of the whole enterprise.

### The "Prudential" Scheme.

It was with considerable interest, therefore, that we learnt a few days ago that a scheme to meet the difficulties of the situation had been suggested by the Prudential Approved Society for Domestic Servants (Women), and we are able as the result of inquiry to give full particulars of it.

The idea of the scheme is founded on the recognition of the fact that in most homes domestic servants are well cared for during short illnesses of not more than, say, six weeks duration, after which if the illness continues the mistress is relieved of her liability by the discharge of the servant. The servant's need is, therefore, for an adequate sickness benefit after the expiration of the first six weeks of a continuous illness, when it is likely that she will not have the advantage of the care and attention that was devoted to her by her mistress during the earlier weeks of her incapacity. It can hardly be argued that the normal sickness benefit of 7s. 6d. a week is adequate to meet the cost of maintenace and the nursing required in the case of a serious illness, though, of course, it is better than nothing, which has, in fact, been the provision of a large number in similar circumstances in the past. Under the Prudential scheme the amount of sickness benefit after the first six weeks of a continuous sickness would be 13s. a week for the next twenty weeks. This is a much more appropriate figure than the normal 7s. 6d., and the consideration for this large additional benefit is that a small reduction will be made in the amount of sickness benefit during the first six weeks of illness to all servants who adopt the scheme.

In the event of the sickness lasting longer than six months the disablement allowance of 5s. a week, provided under the Act, would become payable, and none of the other benefits—medical, maternity, and sanatorium—are affected by the adoption of the scheme.

The essential requirement of actuarial equivalence has been preserved, while at the same time provision has been made for a return to the normal scale of benefits, if so desired, on giving short notice, by making the amounts of sick pay relinquished during the first six weeks of illness vary with the age at date of sickness.

The following table shows the amounts of sickness benefit payable at certain specimen ages, and also exhibits the amount of the normal sick pay relinquished during the first six weeks' illness:—

| Age at Date of Sickness. | Sick Pay. Commencing on fourth day of illness, for any period not exceeding six weeks. | For next twenty weeks. | Relinquished for the first six weeks of illness. |
|---|---|---|---|
| | Per week. s. d. | Per week. s. d. | Per week. s. d. |
| 21 | 5 7 | 13 0 | 1 11 |
| 25 | 5 3 | 13 0 | 2 3 |
| 30 | 5 0 | 13 0 | 2 6 |
| 35 | 4 9 | 13 0 | 2 9 |
| 40 | 4 6 | 13 0 | 3 0 |
| 45 | 4 1 | 13 0 | 3 5 |

It will be seen that a servant falling ill at the age of thirty would stand the chance of losing 15s. during the first six weeks of sickness, but this would be compensated by the gain of £6 10s. if her sickness were to last for another twenty weeks, by the receipt of £13 instead of the normal benefit of £7 10s. So large a difference would at first sight seem almost incredible, but it is justified by the fact that illnesses of short duration are so much more numerous than those that are prolonged for six months or more.

### The Scheme not yet Approved by the Commissioners.

We learn that this scheme was submitted to the Insurance Commissioners two months ago, and that it received favourable consideration, but it has not yet received their official sanction, the reason for the delay being that they are not satisfied, on a technical legal point arising out of the wording of the Act, that they have at present the power to do so. The scheme so exactly meets our view of the requirements of domestic servants that we hope the Commissioners will be able to sanction its approval. If their powers are at present not sufficiently wide for this to be done it would seem to be a matter of imperative necessity that steps should be taken as soon as possible to rectify the deficiency.

There is, fortunately, still sufficient time for the scheme to be approved before the benefits under the Act begin to accrue, provided the Commissioners act promptly; but whether it is sanctioned before January 15 or not, it is evident that the best interests of domestic servants have been considered by the advisers of the Prudential Society.

**NOTE.—Questions and Correspondence on all doubtful points are invited, and should be addressed to the Editor, The Hospital, 28 Southampton Street, Strand, W.C., and marked "Insurance."**

